# A commentary on can ChatGPT assist urologists manage overactive bladder?

**DOI:** 10.1097/JS9.0000000000001261

**Published:** 2024-03-04

**Authors:** Changkun Mao, Tao Zhang

**Affiliations:** Department of Urology, Anhui Provincial Children’s Hospital, Hefei, Anhui, People’s Republic of China

*Dear Editor*,

Leveraging artificial intelligence (AI)-based chatbots, which utilize expansive language models and natural language processing technologies^1^, has evoked widespread interest across various industries globally, including the medical field, since the introduction of ChatGPT by OpenAI in November 2022^2^. We were particularly intrigued by the innovative work of Gao and Feng^3^ in the domain of urology, and we extend our congratulations on their achievements. Their exploration into ChatGPT’s application for assisting in the management of overactive bladder (OAB) not only highlights the potential of ChatGPT in therapeutic decision-making, side effect management, and patient support but also illustrates its value in guiding lifestyle modifications through specific case studies. As pediatric urologists often tasked with managing children with OAB, we are keen to present our perspectives and engage in further discourse on this subject.

Firstly, while the paper demonstrates ChatGPT’s potential utility in managing OAB, evidence of its actual efficacy and safety remains limited. Future research, through clinical trials, case–control studies, or longitudinal studies, is essential to ensure ChatGPT’s effective and safe application in clinical settings. These investigations should encompass diverse patient populations to evaluate the suitability and accuracy of ChatGPT’s recommendations, as well as monitor any potential side effects or misinformation. Moreover, engaging in a feedback loop with medical professionals can further refine ChatGPT’s performance, aligning its recommendations with current clinical guidelines and best practices^4^. Such an integrated approach would deepen our understanding of ChatGPT’s practical value in medical contexts, safeguarding patient safety and enhancing treatment outcomes.

Secondly, despite ChatGPT’s significant potential in aiding medical management, its capabilities are limited in terms of accuracy, reliability, and handling complex medical situations^5^. On the one hand, ChatGPT may struggle to fully comprehend or accurately interpret complex medical terminology and patient records, especially in cases of rare conditions or specific medical scenarios. On the other hand, while its responses are generated from vast datasets, potential biases in training data could lead to recommendations that may not be the most suitable for individual patients. Additionally, delays in updating ChatGPT with the latest medical research and guidelines could impact its ability to provide cutting-edge medical advice. Consequently, any clinical decisions based on ChatGPT’s recommendations should be approached with caution and ultimately reviewed and validated by healthcare professionals^6^.

Lastly, when using AI technologies like ChatGPT to process sensitive health information, adherence to the strictest privacy protection and data security standards is mandatory. This includes compliance with laws and regulations such as the Health Insurance Portability and Accountability Act (HIPAA) and the General Data Protection Regulation (GDPR), ensuring encrypted storage and transmission of patient data, and restricting access to sensitive information^7^. Additionally, patients must be clearly informed about how their data is used and consent obtained explicitly. Ethical considerations must also address potential biases and inequalities introduced by AI technology, ensuring its application does not exacerbate existing disparities in healthcare. The development and application of medical AI should actively undergo ethical review, adhering to medical ethical principles like respect for patient autonomy, non-maleficence, justice, and beneficence.

In summary, this study presents valuable insights and possibilities for ChatGPT’s application in treating OAB. We look forward to future research addressing these issues and further exploring and validating ChatGPT’s efficacy and safety in clinical practice.

## Ethical approval

Not applicable.

## Consent

Not applicable.

## Source of funding

Not applicable.

## Author contribution

T.Z.: writing; C.M.: review, editing, and supervision.

## Conflicts of interest disclosure

The authors declare no conflicts of interest.

## Research registration unique identifying number (UIN)

Not applicable.

## Guarantor

All authors.

## Data available statement

Not applicable.

## Provenance and peer review

Yes, we agree.
